# Identification of the Core Competencies Required in Endodontics for Undergraduate Students in Syrian Dental Schools by Using a Modified Delphi Technique: Prospective Exploratory Survey Study

**DOI:** 10.2196/83799

**Published:** 2026-06-09

**Authors:** Muhammad Salameh, Mayssoon Dashash, Issam Jamous

**Affiliations:** 1 Medical Education Program Syrian Virtual University Damascus, Dimashq Syrian Arab Republic; 2 Department of Endodontics Faculty of Dentistry Al Andalus University Tartus Syrian Arab Republic; 3 Department of Pediatric Dentistry Faculty of Dentistry Damascus University Damascus, Dimashq Syrian Arab Republic; 4 Department of Fixed Prosthodontics Faculty of Dentistry Damascus University Damascus, Dimashq Syrian Arab Republic

**Keywords:** endodontics, Syria, undergraduate curriculum, competency-based medical education, modified Delphi technique, Delphi technique, consultation meeting, root canal treatment

## Abstract

**Background:**

There is a worldwide movement toward competency-based medical education to equip dental students with essential competencies required to meet health care needs. In Syria, dental faculties currently lack a formal competency-based curriculum for endodontics at the undergraduate level. Moreover, the quality of root canal treatment performed by general dentists is frequently described as inadequate or substandard.

**Objective:**

This study aimed to develop a national consensus on the required competencies for undergraduate endodontics in Syria in order to establish a foundation for a standardized national curriculum, which can guide educators in adopting best practices in both dental education and clinical endodontics.

**Methods:**

This study was conducted at Syrian Virtual University between April and June 2025. A modified Delphi technique was used to determine endodontic competencies. Initially, a group of 5 Syrian endodontic consultants identified preliminary competencies. In the first round, 53 experts evaluated these competencies by using a 5-point Likert scale. Based on these results, a second round was conducted with 38 experts. Competencies with a weighted average above 4.20 were considered essential. Data analysis was performed using IBM SPSS package 27, and survey reliability was measured by Cronbach α.

**Results:**

Following the final Delphi round, a set of 31 competencies was established, comprising 9 knowledge, 13 skills, and 9 attitudes competencies. Cronbach α was more than 0.9 in the first and second round. The standard deviation across all questionnaires was low (≤0.85). The standard error was also minimal (≤0.12).

**Conclusions:**

This study identified a set of core endodontic competencies for the undergraduate level in Syria. These competencies are intended to support students in acquiring the required knowledge, skills, and attitudes, and assisting policymakers in implementing competency-based medical education within Syria and similar contexts.

## Introduction

General dentists frequently serve as the primary providers of endodontic treatment for patients requiring such care [1,2]. However, cross-sectional studies often report the quality of root canal treatment as inadequate or below standard [3-7]. This may be attributed to the complexity of root canal procedures or the graduation of practitioners with limited experience [8,9].

The Commission of Dental Accreditation, the Association of Dental Education in Europe, and the European Society of Endodontology (ESE) have all recommended adopting competency-based medical education (CBME) within a comprehensive clinical care environment [8,10,11].

According to the International CBME collaborators, CBME is “an outcome-based approach to the design, implementation, assessment, and evaluation of medical education programs using an organized framework of competencies“ [12]. The goal of CBME is to produce graduates who can effectively incorporate knowledge, skills, and attitudes and apply them clinically to deliver the best patient care [13]. CBME emphasizes outcomes and practice-based learning while considering societal and patient needs by tailoring competency frameworks accordingly [14]. As a result, several countries have adopted CBME into undergraduate medical education [15,16] although its application remains in early stages [16].

In Syria, medical education follows a traditional, time-based approach that emphasizes teacher-centered learning, hospital-based training, and opportunistic learning methods [17]. To our knowledge, there is no existing literature detailing the design and implementation of curricula in Syrian medical schools. Typically, curriculum development and review are undertaken by a small group of academic members without formal consensus from all faculty members before final approval by the faculty and university councils [18].

Competency-based education is a fundamental part of the academic curriculum, as it measures and verifies the attainment of the intended program learning outcomes [19]. Therefore, the initial step in implementing a CBME is to identify the required competencies [20]. This method offers medical students focused and well-structured training in endodontics, ensuring they acquire the necessary knowledge, skills, and attitudes to provide effective patient care. Within this framework, this study aims to establish a national consensus on the core competencies required for undergraduate endodontics curricula in Syria.

CBME considers competencies as the key outcomes that should guide curriculum development at every stage, including implementation, assessment, and evaluation [21-23]. To embrace CBME and adequately prepare medical students for clinical practice, educators usually rely on an organized national or international competency framework that describes the abilities physicians need to meet patients’ and societal demands [24].

The Delphi technique, a widely used consensus method for identifying health professional competencies, has been applied independently [25] or alongside focus groups [26] and consultation meetings [27]. In this study, a competency framework for undergraduate endodontics was developed using a combination of the Delphi technique and consultation meetings to identify essential competencies. Therefore, this study aims to develop a national consensus on essential undergraduate endodontic competencies in Syria by using a modified Delphi method.

## Methods

### Overview

This exploratory qualitative study was undertaken at the Syrian Virtual University from April to June 2025. The modified Delphi technique was used to reach a consensus on the competencies for the undergraduate endodontics curriculum.

The consultation group included 5 Syrian members holding a PhD in endodontics and working in academic roles at various universities. Additionally, experts were either Syrians or held equivalent qualifications, possessed at least a specialist certificate (Syrian board) in endodontics, and they were currently actively involved in clinical, academic, and/or administrative practice inside or outside Syria.

The principal researcher (MS) developed a preliminary list of endodontic competencies based on a literature review [28-30]. Individuals' consultation meetings were then held via telephone or social media platforms, lasting at least 15 minutes each, and conducted at least twice with each consultant. These meetings reviewed the initial list of competencies, allowing for modifications and additions. The consultation meetings produced a final list of 54 competencies divided into 19 knowledge, 21 skills, and 14 attitude items. A structured questionnaire was developed using Google Forms, consisting of six sections: (1) an introductory letter explaining the study’s objectives, confidentiality assurance, participation instructions, and consent acknowledgement; (2) demographic data, including name, age, gender, nationality, workplace, years of experience, academic qualifications, and practice type; (3,4,5) the initial lists of knowledge, skills, and attitudes competencies in the third, fourth, and fifth sections, respectively; and (6) a free-text section for respondents to suggest additional competencies (Multimedia Appendix 1).

The Delphi methodology was conducted in 2 rounds to identify the essential endodontic competencies for undergraduate dental students in Syrian dental schools. The principal researcher MS invited 98 endodontists who met the expert panel criteria to participate, with 73 accepting the invitation. To ensure a national consensus, these experts represented dental schools from both public and private universities across Syria. Participants rated each competency on a 5-point Likert scale reflecting its importance: 1 (not important at all), 2 (not important), 3 (unsure), 4 (important), and 5 (very important). Consensus was defined as over 80% agreement or a mean score above 4.20 [25,31].

Strict deadlines were set to maintain study momentum while allowing participant flexibility with accountability to ensure a smooth and efficient progression through its phases [32]. The first Delphi round lasted 3 weeks, starting on May 2, 2025, with reminders sent to nonrespondents on the 5th, 10th, and 15th. Competencies scoring above 4.20 in the first round, along with those suggested through free-text comments, were included in the second round. The second round had a 2-week deadline, with reminders on days 5 and 10 for those who had not responded.

Data analysis was performed using the SPSS package (version 27; IBM Corp). The survey's reliability was assessed using Cronbach α, and descriptive statistics, including the mean, standard deviation, and standard error, were calculated.

### Ethical Considerations

Ethical approval was obtained from the research committee of the Syrian Virtual University (455/0, dated April 27, 2025). Informed consent was obtained from all participating consultants and experts before the study.

## Results

The characteristics of the participants in both the first and second Delphi rounds are shown in Table 1. Following the consultation meetings, a preliminary list of 54 competencies was established, comprising 19 (35.2%) knowledge competencies, 21 (38.8%) skills competencies, and 14 (26%) attitudes competencies (Multimedia Appendix 1). In the first Delphi round, 53 out of the 73 participants completed the survey, yielding a response rate of 72.6%.

**Table 1:** Characteristics of the respondents in the two Delphi rounds.

Characteristics			Round 1, n (%)		Round 2, n (%)
Sample					
	Participants	73 (100)		73 (100)	
	Respondents	53 (72.6)		38 (52)	
Gender					
	Male	40 (75.5)		27 (71)	
	Female	13 (24.5)		11 (29)	
Qualifications					
	PhD	38 (71.7)		27 (71)	
	Master	13 (24.5)		9 (23.7)	
	Other	2 (3.8)		2 (5.3)	
Years of experience					
	≤ 5 y	6 (11.3)		2 (5.3)	
	6-10 y	13 (24.5)		9 (23.7)	
	11-15 y	10 (18.8)		7 (18.4)	
	16-20 y	7 (13.2)		9 (23.7)	
	21-25 y	10 (18.9)		4 (10.5)	
	˃25 y	7 (13.2)		7 (18.4)	
Type of work					
	Academic only	14 (26.4)		14 (36.8)	
	Clinical only	11 (20.7)		9 (23.7)	
	Academic & clinical	22 (41.5)		10 (26.3)	
	Academic, clinical, & administrative	6 (11.3)		5 (13.2)	

In the first Delphi round, 34 competencies were approved, consisting of 10 knowledge, 15 skills, and 9 attitudes. Additionally, participants suggested 4 new competencies, 1 knowledge, and 3 attitudes, bringing the total to 38 for the second round (Multimedia Appendix 2). These newly suggested competencies were evaluated by participants using the same criteria as in the first round. In the second Delphi round, 38 out of 73 experts completed the survey, resulting in a 52% response rate. Table 1 presents the characteristics of the second-round participants. Of the 38 competencies, 31 (81.5%) received mean ratings above 4.20. These were distributed across the domains as follows: 9 (29%) knowledge, 13 (42%) skills, and 9 (29%) attitude (Table 2). In this round, competencies were ranked based on experts’ weighted responses.

**Table 2:** Final competencies in endodontics for the undergraduate level.

Competency		Mean	SE	SD
Knowledge				
	Diagnostic investigations, 2D and 3D radiography	4.68	.083	.518
	Dental materials, biomaterials, and adjunct therapies applied to the management of endodontics	4.53	.080	.499
	Oral and dental diseases related to endodontics	4.42	.079	.494
	Basic sciences and their relationship to endodontics	4.39	.078	.489
	Pharmacology and therapeutics as applied to the management of dental patients	4.39	.094	.587
	Principles of general medicine and surgery applied to the management of dental patients (including endodontics)	4.24	.093	.582
	Principles of optical magnification	4.24	.107	.666
	Principles of management of immature teeth	4.21	.098	.614
	Treatment options for a postendodontic problem	4.21	.111	.694
Skill				
	Management of endodontic emergencies	4.58	.079	.494
	Reliable and appropriate isolation	4.53	.080	.499
	Use various intraoral anesthesia techniques and pain control	4.50	.080	.500
	Establishing a reliable root canal irrigation protocol	4.50	.088	.550
	Diagnose and differentiate odontogenic pain or lesions	4.47	.080	.499
	Reaching a diagnosis and identifying possible differential diagnoses, including their etiology	4.42	.125	.782
	Performing high-quality endodontic treatments on extracted or simulated (Acrylic Blocks) teeth of various types	4.42	.114	.712
	Perform high-quality clinical endodontic treatment using conventional methods for easy or moderately difficult teeth (according to the American Association of Endodontics classification)	4.42	.087	.544
	Develop a treatment plan and communicate this to the patient	4.39	.101	.630
	Restoration of endodontically treated teeth, including root canal posts	4.37	.100	.625
	Conducting a comprehensive clinical examination of a patient presenting with an endodontic-related problem	4.34	.123	.770
	Assess the case difficulty and request a consultation or refer the patient	4.34	.123	.770
	Conducting a detailed general and dental history	4.24	.107	.666
Attitudes				
	Respect patient privacy and confidentiality	4.66	.076	.474
	Self-efficacy	4.42	.087	.544
	Time and priority management	4.42	.087	.544
	Commitment to lifelong learning	4.37	.100	.625
	Compliance with local regulations regarding infection control, radiation protection, record keeping, and documentation	4.37	.100	.625
	Handling medical waste	4.37	.093	.581
	Communicate verbally and in writing with dental and medical colleagues	4.34	.112	.699
	Communicate effectively with the patient or their family	4.34	.099	.619
	Informed consent	4.34	.099	.619

Based on the results of the second Delphi round, the final list of competencies for the undergraduate endodontics curriculum was finalized (Table 2).

The standard deviation for all questionnaire items was low, not exceeding 0.85, and the standard error was also minimal, remaining below 0.12. These values indicate a close alignment between experts' individual ratings and the overall mean. Additionally, Cronbach α exceeded 0.9 for both the first and second rounds, demonstrating a high reliability level of internal consistency reliability (Table 3).

**Table 3:** Cronbach α results in both Delphi rounds.

Domain of competency	Total	Attitude	Skill	Knowledge
First round	0.901	0.783	0.828	0.863
Second round	0.909	0.832	0.845	0.749

## Discussion

### Principal Findings

The findings of this study support a national consensus among experts regarding the core competencies required in endodontics at the undergraduate level in Syria. A set of 31 competencies was established, comprising 9 knowledge, 13 skills, and 9 attitudes.

There is no universally accepted definition of endodontic competency for the new general dentist [33]. Moreover, there are currently no national or international guidelines for undergraduate endodontic education specifying the essential materials and equipment that students should be competent in using [34]. This study was conducted to address that gap.

Developing a national or international framework for undergraduate endodontic education can help educators reach consensus and implement best practices in education and endodontics [29]. Clearly defining the required materials and equipment is crucial for standardized practical training [34]. The primary goal of undergraduate endodontic education is to produce competent clinicians who understand the basic science of endodontics and recognize limitations, ensuring safe general dental practice. Clinical training should also emphasize the appropriate use of treatment consent and case difficulty assessment [29], which were listed in the core competencies in this study.

While CBME shows promise, implementation faces real challenges: resistance to change, budget constraints, and difficulty assessing competencies like attitudes. To succeed, institutions should involve stakeholders in redesign efforts, pursue external funding, and employ diverse assessment methods—including observation and simulation—to evaluate competency development more comprehensively [35].

According to a systematic review, questionnaires are the most widely used tool for identifying required competencies in medical education worldwide [24]. This aligns with the approach taken in this study.

The Delphi technique is among the most widely used techniques for defining competencies in medical education, offering many advantages over other decision-making methods. It stimulates a sense of responsibility and supports acceptance of consensus outcomes, minimizing bias from dominant individuals. This enhances the reliability and validity of the required core competencies [15,25]. A modified Delphi method incorporating consultation meetings with endodontic consultants was preferred for its accuracy and efficiency in identifying initial competencies and selecting experts. These meetings were conducted individually to prevent the influence of more senior or dominant consultants on others' opinions. The survey’s results validated the consultation group, as all 27 excluded competencies were rated within the ”important“ range, except 3, which were in the ”unsure“ range. Notably, none of the 4 competencies proposed by participants in the first round were approved in the second round.

The diverse backgrounds of the participants contributed to a well-rounded and unbiased consensus on core endodontics competencies tailored to the needs of a Syrian society. Despite this diversity, the low standard deviation across both questionnaires indicates a strong agreement and minimal variation in opinions. The small standard error demonstrates that the mean ratings of the experts’ opinions closely matched the approved means (Table 2). Finally, Cronbach α results confirm the internal consistency and reliability of the survey in both rounds (Table 3).

Medical literature suggests a sample size of approximately 20 individuals for this type of study [36], though larger sample sizes tend to yield more accurate results [37]. In this study, the sample size was sufficiently large, with at least 38 participants, whereas most similar studies typically involve 15-30 participants [38].

While multi-round studies with large samples often struggle with declining response rates—potentially undermining consensus credibility—our study achieves more than 70% response rate (38 of 53 participants), which meets or exceeds the standard benchmark many researchers consider essential for robust consensus findings [39,40].

The use of an electronic questionnaire via Google Forms was chosen to save time, effort, and cost. Nair et al [41] suggested strategies to improve survey response rates, including sending reminders at specific time intervals, an approach that was successfully implemented in this study, resulting in significant increases in responses after each reminder. Two rounds were adopted because no new competencies were added in the second round, and less than 9% of competencies were excluded. Jünger et al [42] stated in his methodological study that most Delphi-based research is satisfied with 2 rounds, which aligns with the procedure followed in this study. The threshold for accepting a competency as essential (must know) was set at a mean score above 4.20. Jünger et al [42] explained that the researcher determines this cutoff based on the nature and objectives of the research. This approach is consistent with similar studies identifying core competencies within specific medical specialties [18,32,40].

Twenty competencies, representing 37% of those proposed by the researcher and consultants, were excluded after the first round—a notably large proportion. This is because the primary references used to develop these competencies, especially as reported by Baaij et al [28] from the ESE, do not differentiate between the basic competencies required in undergraduate versus postgraduate endodontics, as the vast majority of European countries do not recognize endodontics as a separate, independent specialty, but instead, they consider it as part of a general dentist's responsibilities [43,44]. Furthermore, these competencies were intended as recommendations for undergraduate endodontics curricula rather than essential competencies that students should master before graduation.

The [28] framework established 3 competency levels: be competent, have knowledge, and be familiar with. This study mirrors most ”be competent“ items, with 2 notable exceptions: managing uncertainty and maintaining pulp vitality were rated as “important“ by our expert panel. This distinction reflects expert opinion that these advanced competencies fall outside the general dentistry scope and are more appropriately reserved for endodontic specialists.

It is worth noting that 85% of the excluded competencies were still rated within the “important” category. These can be interpreted as important competencies for the student, and ”it is good to know,“ but they are not essential to master them at the undergraduate level.

By the end of the second round, 7 competencies were further excluded, including all 4 additional competencies suggested by participants. The remaining 3 competencies were close to the inclusion threshold for core competencies (Table 2).

Despite extensive research on identifying competencies in endodontics, no prior studies have directly addressed this topic comprehensively [29]. However, several institutions involved in medical and dental education such as the American Association of Endodontics, Association of Canadian Faculties of Dentistry, Canadian Medical Education Directives for Specialists, Commission of Dental Accreditation of Canada, ESE, General Dental Council, and the Saudi Commission for Health Specialties have provided guidelines for designing undergraduate or residency-level endodontic curricula.

The ESE recommended essential undergraduate endodontic competencies [28]. While this study identifies competencies that align closely with ESE's recommendations, the division of competency domains and the method of determining the competencies differ significantly. The ESE did not use a questionnaire nor distinguish between undergraduate and postgraduate endodontics, reflecting the absence of endodontic specialization in most European countries.

The study has some limitations that should be considered. Involving additional stakeholders such as students, patients, and health care or education administrators, through interviews rather than questionnaires, could enrich the findings but would also broaden the study's scope and complicate data analysis. So it would be preferable to build upon the results of this study, which relied on the endodontists’ opinions, with further studies targeting the views of other stakeholders to determine their needs and perspectives based on these findings.

Additionally, assigning numerical values 1-5 to responses may not capture the nuances of expert input; for example, a professor with more than 30 years of experience carries the same weight as a recent graduate. Nevertheless, including a range of perspectives across generations is important to ensure balanced and unbiased results. The high degree of harmony in opinions among the experts’ panel indicates an implicit agreement on the core competencies that a graduate should possess as a graduation requirement.

### Conclusion

This study identifies the essential endodontics competencies for undergraduate education in Syria by using a modified Delphi method. Further studies involving a broader range of stakeholders should be considered to confirm the results. Additionally, developing an international framework for undergraduate endodontics competencies through collaboration with researchers worldwide would be beneficial. The final list of the identified competencies provides a framework for developing a formal competency-based endodontics curriculum for the undergraduate stage, which can assist students in achieving the required standards and advancing the adoption of CBME in Syria and other similar settings.

A similar study must be conducted to identify the core competencies required for the postgraduate stage in endodontics in Syria. This would clearly differentiate between undergraduate and postgraduate endodontic requirements.

This study employs a modified Delphi method to identify essential endodontics competencies for undergraduate dental education in Syria. The resulting competency framework has been established as a foundation for developing a competency-based endodontics curriculum that supports students in meeting established standards and advancing CBME adoption across Syria and comparable contexts.

To strengthen these findings, broader stakeholder engagement is needed to validate the competency list. It is recommended that the research scope be expanded by including diverse stakeholders to confirm and refine results. Additionally, an international competency framework should be established through collaborative efforts with global researchers to ensure consistency and align best practices across regions.

Implementation of this framework should incorporate continuous assessment strategies, including clinical observation; multi-source feedback from patients, peers, and students; and learner self-assessment to monitor competency development and identify areas for improvement. These varied assessment methods enable timely intervention and support the transition toward CBME in Syria and similar settings.

Finally, a parallel study should be conducted to identify core competencies for postgraduate endodontics training in Syria. This research would create clear differentiation between undergraduate and graduate-level requirements, establishing a coherent progression pathway for dental professionals and ensuring comprehensive competency development across all training stages.

## Data Availability

The datasets generated and analyzed during this study are not publicly available due to participant confidentiality and institutional restrictions. However, deidentified data may be made available from the corresponding author upon reasonable request and with appropriate ethical clearance.

## Appendix

Multimedia Appendix 1 First-round questionnaire: identification of core competencies required in endodontics for undergraduate students in Syrian dental schools, using a modified Delphi technique.

Multimedia Appendix 2 Second-round questionnaire: identification of core competencies required in endodontics for undergraduate students in Syrian dental schools, using a modified Delphi technique.

## Abbreviations

CBME: competency-based medical education
ESE: European Society of Endodontology

## References

[1] Alrahabi M, Ahmad M (2015). Knowledge regarding technical aspects of non-surgical root canal treatment in Al-Madinah Al-Munawarah private dental centers. Saudi Endod J.

[2] Dietz GC Sr, Dietz GC Jr (1992). The endodontist and the general dentist. Dental Clinics of North America.

[3] Al-Obaida M, Alwehaiby K, Al-Hindi O, Merdad K, Al-Madi E (2020). Radiographic evaluation of the technical quality of root canal filling in Riyadh government and private hospitals. Saudi Endod J.

[4] Alrahabi M, Younes H (2016). A cross-sectional study of the quality of root canal treatment in Al-Madinah Al-Munawwarah. Saudi Endod J.

[5] Di Filippo G, Sidhu SK, Chong BS (2014). Apical periodontitis and the technical quality of root canal treatment in an adult sub-population in London. Br Dent J.

[6] Chueh L, Chen S, Lee C, Hsu Y, Pai S, Kuo M, Chen C, Duh B, Yang S, Tung Y, Hsiao CK (2003). Technical quality of root canal treatment in Taiwan. Int Endod J.

[7] Boucher Y, Matossian L, Rilliard F, Machtou P (2002). Radiographic evaluation of the prevalence and technical quality of root canal treatment in a French subpopulation. Int Endod J.

[8] Koch M, Wolf E, Tegelberg A, Petersson K (2015). Effect of education intervention on the quality and long-term outcomes of root canal treatment in general practice. Int Endod J.

[9] Qualtrough AJE (2014). Undergraduate endodontic education: what are the challenges?. Br Dent J.

[10] (2018). Commission on Dental Accreditation Standards for Dental Education Programs. Commission on Dental Accreditation.

[11] Levin L, Halperin-Sternfeld M (2013). Tooth preservation or implant placement: a systematic review of long-term tooth and implant survival rates. J Am Dent Assoc.

[12] Frank JR, Snell LS, Cate OT, Holmboe ES, Carraccio C, Swing SR, Harris P, Glasgow NJ, Campbell C, Dath D, Harden RM, Iobst W, Long DM, Mungroo R, Richardson DL, Sherbino J, Silver I, Taber S, Talbot M, Harris KA (2010). Competency-based medical education: theory to practice. Med Teach.

[13] ten Cate O, Scheele F (2007). Viewpoint: competency-based postgraduate training: can we bridge the gap between theory and clinical practice?. Academic Medicine.

[14] Frank JR, Mungroo R, Ahmad Y, Wang M, De Rossi S, Horsley T (2010). Toward a definition of competency-based education in medicine: a systematic review of published definitions. Med Teach.

[15] Shah S, McCann M, Yu C (2020). Developing a national competency-based diabetes curriculum in undergraduate medical education: a Delphi study. Can J Diabetes.

[16] Veale P, Busche K, Touchie C, Coderre S, McLaughlin K (2019). Choosing our own pathway to competency-based undergraduate medical education. Acad Med.

[17] Souliman M, Dashash M (2023). Reasons for failure to change Syrian medical curricula: a qualitative descriptive study. Azerbaijan Medical Journal.

[18] Zoukar I, Dashash M (2023). Using a modified Delphi method for identifying competencies in a Syrian undergraduate neonatology curriculum. Matern Child Health J.

[19] Soundariya K, Nishanthi A, Mahendran R, Vimal M (2025). Evaluation of competency-based medical education (CBME) curriculum implementation for phase II medical undergraduates: a qualitative study. J Adv Med Educ Prof.

[20] Leung WC (2002). Competency based medical training: review. BMJ.

[21] Danilovich N, Kitto S, Price DW, Campbell C, Hodgson A, Hendry P (2021). Implementing competency-based medical education in family medicine: a narrative review of current trends in assessment. Fam Med.

[22] Caccia N, Nakajima A, Kent N (2015). Competency-based medical education: the wave of the future. J Obstet Gynaecol Can.

[23] Hawkins Richard E, Welcher Catherine M, Holmboe Eric S, Kirk Lynne M, Norcini John J, Simons Kenneth B, Skochelak Susan E (2015). Implementation of competency-based medical education: are we addressing the concerns and challenges?. Med Educ.

[24] Alharbi N (2024). Evaluating competency-based medical education: a systematized review of current practices. BMC Med Educ.

[25] Dashash M, Almasri B, Takaleh E, Halawah A, Sahyouni A (2020). Educational perspective for the identification of essential competencies required for approaching patients with COVID-19. East Mediterr Health J.

[26] Geng Y, Zhao L, Wang Y, Jiang Y, Meng K, Zheng D (2018). Competency model for dentists in China: results of a Delphi study. PLoS One.

[27] Benzian H, Greenspan J, Barrow J, Hutter J, Loomer P, Stauf N, Perry Da (2015). A competency matrix for global oral health. Journal of Dental Education.

[28] Baaij A, Kruse C, Whitworth J, Jarad F (2024). European Society of Endodontology undergraduate curriculum guidelines for endodontology. Int Endod J.

[29] Algahtani FN, Barakat RM, Almohareb RA, Alqarni L, Alqabbani A, Almadi E (2022). The objectives and instructional design of undergraduate endodontic program: multicenter cross-sectional study in Saudi Arabia. BMC Med Educ.

[30] (2016). ACFD educational framework for the development of competency in dental programs. Association of Canadian Faculties of Dentistry.

[31] Guan L, Gao P, Liu S, Liu Y, Li X, Liu F, Mao Z, Lu Y, Xiang H (2019). Development of a global health bachelor curriculum in China: a Delphi study. BMJ Open.

[32] Zoukar I, Zoukar S, Muad M, Dashash M (2025). Establishing core competencies for neonatology fellowship training in Syria: a modified Delphi study approach. BMC Med Educ.

[33] Dutner JM, Sidow SJ, Cervero RM, Soh M (2024). Endodontic competence of the new general dentist as conceptualized by predoctoral educators: a qualitative exploration. J Dent Oral Epidemiol.

[34] Algahtani FN, Barakat RM, Alqarni LM, Alqabbani AF, Alkadi MF, Almohareb RA (2023). Undergraduate endodontic training and its relation to contemporary practice: multicenter cross-sectional study in Saudi Arabia. Int J Clin Pract.

[35] Ramanathan R, Shanmugam J, Gopalakrishnan S, Palanisamy K, Narayanan S (2022). Challenges in the implementation of competency-based medical curriculum: perspectives of prospective academicians. Cureus.

[36] Dunn WR, Hamilton D D, Harden R M (1985). Techniques of identifying competencies needed of doctors. Med Teach.

[37] Hasson F, Keeney S, McKenna H (2000). Research guidelines for the Delphi survey technique. J Adv Nurs.

[38] Sahyouni A, Zoukar I, Dashash M (2024). Evaluating the effectiveness of an online course on pediatric malnutrition for Syrian health professionals: qualitative delphi study. JMIR Med Educ.

[39] Keeney S, McKenna H, Hasson F (2011). The Delphi Technique in Nursing and Health Research.

[40] Giannarou L, Zervas E (2014). Using Delphi technique to build consensus in practice. IJBSAM.

[41] Nair CS, Wayland C, Soediro S (2005). Evaluating the student experience: a leap into the future. https://tinyurl.com/4exwjp65.

[42] Jünger S, Payne SA, Brine J, Radbruch L, Brearley SG (2017). Guidance on Conducting and REporting DElphi Studies (CREDES) in palliative care: recommendations based on a methodological systematic review. Palliat Med.

[43] García-Espona I, García-Espona E, Alarcón JA, Fernández-Serrano J (2023). European inequalities and similarities in officially recognized dental specialties. BMC Oral Health.

[44] Al Raisi H, Dummer PMH, Vianna ME (2019). How is endodontics taught? A survey to evaluate undergraduate endodontic teaching in dental schools within the United Kingdom. Int Endod J.

