# Supplementary material for: Identification of the Core Competencies Required in Endodontics for Undergraduate Students in Syrian Dental Schools by Using a Modified Delphi Technique: Prospective Exploratory Survey Study
Source: Interact J Med Res. 2026 Jun 9;15:e83799. doi: 10.2196/83799 (PMC13291728; doi:10.2196/83799)
Supplement: Multimedia Appendix 1 [file ijmr_v15i1e83799_app1.docx]

**First Round questionnaire for Identification of Core Competencies Required in Endodontics for Undergraduate Students in Syrian Dental Schools Using a Modified Delphi Technique**

**A. Introduction**

**Dear colleagues**

I am Dr. Muhammad Salameh, an Associate Professor of Endodontics, at Al-Andalus University, Syria.

I am sending you this questionnaire for the first round. It aims to identify the key competencies required for endodontics at the undergraduate level in Syrian dental schools.

The Delphi technique will be used to determine the consensus among colleagues in identifying the key competencies required in endodontics. This technique requires several rounds to reach consensus, which means that the competency will achieve 80% of agreements or the mean for responses be more than 4.20.

The answers to the competencies are based on a five-point Likert scale:

1. Not important at all.
2. Not important.
3. Unsure.
4. Important.
5. Very important.

At the end of the questionnaire, a space is made for colleagues to add any competency they believe is important, which was not included in the questionnaire, or to modify any competency included in the questionnaire that is ambiguous or unclear.

The questionnaire is confidential, and only the researcher will have access to its results.

If you have any question, please contact me on the following mobile number: +963944273017 or via WhatsApp on the same number.

I am pleased and honored by your agreement to participate in this study.

**B. Personal Information**,

Name:

Age: 30 yrs or less, 31-40 yrs, 41-50 yrs, 51-60 yrs, or more than 60 yrs.

Gender: male or female.

Academic Qualifications: Master's, PhD, or other.

Workplace: Public University, Private University, Private Clinic, and/or Other.

Type of practice: Clinical, Academic, and/or Administrative.

Years of experience after obtaining the specialty: 5yrs or less, 6-10yrs, 11-15yrs, 16-20yrs, 21-25yrs, or more than 25yrs.

Country of work:

**Key competencies required for undergraduate endodontics:**

**C. Domain of knowledge**: The graduate should have a sound knowledge of the following tips:

1. Basic sciences (dental histology, oral and dental physiology, biochemistry, immunology, head and neck anatomy, dental anatomy, and microbiology) and their relationship to endodontics.
2. Oral and dental diseases related to endodontics.
3. Principles of general medicine and surgery applied to the management of dental patients (including endodontics).
4. Pharmacology and therapeutics as applied to the management of dental patients.
5. Dental materials, biomaterials, and adjunct therapies applied to the management of endodontics
6. Epidemiology, public health measures, and biostatistics.
7. Basic computer skills.
8. Fundamentals of scientific research and publishing.
9. Principles of ergonomics.
10. Diagnostic investigations, 2D and 3D radiography.
11. Principles of optical magnification
12. Fundamentals of management of dentofacial trauma.
13. Principles of management of immature teeth
14. Principles of regenerative endodontics.
15. Principles of dealing with mishaps of endodontic procedures.
16. Treatment options for a patient with a post-endodontic problem
17. Indications and contraindications for endodontic Surgery and its complications
18. Follow-up of patients after endodontic surgery
19. Fundamentals of managing high-risk (or compromised) patients in the dental clinic.

**D. Domain of ​​skills**: The undergraduate should be competent at:

1. conducting a detailed general and dental history.
2. Conducting a comprehensive clinical examination of a patient presenting with an endodontic-related problem.
3. Reaching a diagnosis and identifying possible differential diagnoses, including their etiology.
4. Diagnose and differentiate odontogenic pain or lesions.
5. Assess the Case Difficulty and request a consultation or refer the patient (according to Case Difficulty Assessment of the American Association of Endodontics AAE).
6. Monitor and evaluate the outcome of endodontic treatment.
7. Develop a treatment plan and communicating this to the patient.
8. Performing procedures to retain the vitality of all or part of the dental pulp.
9. Use various intraoral anesthesia techniques and pain control.
10. Management of endodontic emergencies.
11. Establishing a reliable root canal irrigation protocol.
12. Performing high-quality endodontic treatments on extracted or simulated (Acrylic Blocks) teeth of various types.
13. Perform high-quality clinical endodontic treatment using conventional methods for easy or moderately difficult teeth (according to the AAE classification).
14. Perform high-quality endodontic treatment using conventional methods for difficult teeth (according to the AAE classification).
15. Use of certain rotary instrument systems in canal shaping.
16. Apply thermal (warm) obturation techniques.
17. Use of ultrasonic instruments in endodontics.
18. Restoration of endodontically treated teeth, including root canal posts.
19. Tooth bleaching.
20. Reliable and appropriate isolation.
21. Photography and documentation.

**E.** **Domain of ​​attitude:** The graduate should have the following Attitude

1. Communicate verbally and in writing with dental and medical colleagues.
2. Communicate effectively with the patient or their family.
3. Informed consent (explaining the treatment plan to the patient and obtaining their consent).
4. Emotional Intelligence (the ability to understand one's own and others' emotions and manage them in positive ways to relieve stress, communicate effectively, empathize with others, and overcome challenges and conflicts).
5. Self-Efficacy (the belief and confidence that the doctor will be able to successfully perform assigned tasks).
6. Critical Thinking.
7. Commitment to lifelong learning.
8. Compliance with local regulations regarding infection control, radiation protection, record keeping, and documentation.
9. Adoption of a dental concept based on the best and most up-to-date evidence.
10. Ability to deal with complexity and uncertainty.
11. Effective in Teamwork.
12. Respect patient privacy and confidentiality.
13. Time and priority management.
14. Handling with medical waste.

**F. Open Question**

Kindly, add any competency that you believe was not included in the questionnaire, or modify any competency included in the questionnaire (that is ambiguous or unclear). Or any other thought.

Table 1Reliability Statistics: Cronbach's Alpha of first round

| Total | Attitude | Skill | Knowledge | Domain of competency |
| --- | --- | --- | --- | --- |
| 54 | 14 | 21 | 19 | No of competencies |
| 0.901 | 0.783 | 0.828 | 0.863 | Cronbach's Alpha |

Table 2. Qualitative Interpretation of 5-Point Likert Scale Measurements

| Likert scale description | Likert scale | Likert scale interval | Agreement rate % |
| --- | --- | --- | --- |
| Not important at all | 1 | 1-1.80 | 0-20 |
| Not Important | 2 | 1.81-2.60 | 20.25- 40 |
| Unsure | 3 | 2.61- 3.40 | 40.25- 60 |
| Important | 4 | 3.41- 4.20 | 60.25- 80 |
| Very Important | 5 | 4.21- 5 | 80.25- 100 |
