# Supplementary material for: Identification of the Core Competencies Required in Endodontics for Undergraduate Students in Syrian Dental Schools by Using a Modified Delphi Technique: Prospective Exploratory Survey Study
Source: Interact J Med Res. 2026 Jun 9;15:e83799. doi: 10.2196/83799 (PMC13291728; doi:10.2196/83799)
Supplement: Multimedia Appendix 2 [file ijmr_v15i1e83799_app2.docx]

**Second Round questionnaire for Identification of Core Competencies Required in Endodontics for Undergraduate Students in Syrian Dental Schools Using a Modified Delphi Technique**

**A. Introduction**

Dear Collogues

I am grateful for your participating in first round of the questionnaire; therefore, I present to you a summary of the results of that one: 34 competencies (from 54) were approved as key competencies in undergraduate endodontics: 10 competencies in the knowledge domain, 15 competencies in the skill domain, and 9 competencies in the attitude domain. Four new competencies were proposed: 1 in the knowledge domain and 3 in the attitude domain. Please, we are going to conduct a second round of the questionnaire, based on the Delphi technique requirements, which will include the competencies approved in the first round, in addition to the new proposed competencies.

The answers to the competencies are based on a five-point Likert scale:

1. Not important at all.
2. Not important.
3. Unsure.
4. Important.
5. Very important.

At the end of the questionnaire, a space is made for colleagues to add any competency they believe is important, which was not included in the questionnaire, or to modify any competency included in the questionnaire that is ambiguous or unclear.

The questionnaire is confidential, and only the researcher will have access to its results.

If you have any question, please contact me on the following mobile number: +963944273017 or via WhatsApp on the same number.

I am pleased and honored by your agreement to participate in this study.

**B. Personal Information**,

Name:

Age: 30 yrs or less, 31-40 yrs, 41-50 yrs, 51-60 yrs, or more than 60 yrs.

Gender: male or female.

Academic Qualifications: Master's, PhD, or other.

Workplace: Public University, Private University, Private Clinic, and/or Other.

Type of practice: Clinical, Academic, and/or Administrative.

Years of experience after obtaining the specialty: 5yrs or less, 6-10yrs, 11-15yrs, 16-20yrs, 21-25yrs, or more than 25yrs.

Country of work:

**Key competencies required for undergraduate endodontics:**

**C. Domain of knowledge**: The graduate should have a sound knowledge of the following tips:

1. Basic sciences (dental histology, oral and dental physiology, biochemistry, immunology, head and neck anatomy, dental anatomy, and microbiology) and their relationship to endodontics.
2. Oral and dental diseases related to endodontics.
3. Principles of general medicine and surgery applied to the management of dental patients (including endodontics).
4. Pharmacology and therapeutics as applied to the management of dental patients.
5. Dental materials, biomaterials, and adjunct therapies applied to the management of endodontics
6. Diagnostic investigations, 2D and 3D radiography.
7. Principles of optical magnification.
8. Principles of management of immature teeth.
9. Treatment options for a patient with a post-endodontic problem.
10. Fundamentals of managing high-risk (or compromised) patients in the dental clinic.
11. Fundamentals of Artificial Intelligence in Endodontics

**D. Domain of ​​skills**: The undergraduate should be competent at:

1. conducting a detailed general and dental history.
2. Conducting a comprehensive clinical examination of a patient presenting with an endodontic-related problem.
3. Reaching a diagnosis and identifying possible differential diagnoses, including their etiology.
4. Diagnose and differentiate odontogenic pain or lesions.
5. Assess the Case Difficulty and request a consultation or refer the patient (according to Case Difficulty Assessment of the American Association of Endodontics AAE).
6. Monitor and evaluate the outcome of endodontic treatment.
7. Develop a treatment plan and communicating this to the patient.
8. Use various intraoral anesthesia techniques and pain control.
9. Management of endodontic emergencies
10. Establishing a reliable root canal irrigation protocol.
11. Performing high-quality endodontic treatments on extracted or simulated (Acrylic Blocks) teeth of various types.
12. Perform high-quality clinical endodontic treatment using conventional methods for easy or moderately difficult teeth (according to the AAE classification).
13. Restoration of endodontically treated teeth, including root canal posts.
14. Reliable and appropriate isolation.
15. Photography and documentation.

**E.** **Domain of ​​attitude:** The graduate should have the following Attitude

1. Communicate verbally and in writing with dental and medical colleagues.
2. Communicate effectively with the patient or their family.
3. Informed consent (explaining the treatment plan to the patient and obtaining their consent).
4. Self-Efficacy (the belief and confidence that the doctor will be able to successfully perform assigned tasks).
5. Commitment to lifelong learning.
6. Compliance with local regulations regarding infection control, radiation protection, record keeping, and documentation.
7. Respect patient privacy and confidentiality.
8. Time and priority management.
9. Handling with medical waste.
10. Self-assessment and assessment of others.
11. Ability to communicate effectively in English Language
12. Expense management.

**F. Open Question**

Kindly, add any competency that you believe was not included in the questionnaire, or modify any competency included in the questionnaire (that is ambiguous or unclear). Or any other thought.

Table 1Reliability Statistics: Cronbach's Alpha of second round

| Total | Attitude | Skill | Knowledge | Domain of competency |
| --- | --- | --- | --- | --- |
| 38 | 12 | 15 | 11 | No of competencies |
| 0.909 | 0.832 | 0.845 | 0.749 | Cronbach's Alpha |
